# Antibacterial Activities of Acetic Acid against Major and Minor Pathogens Isolated from Mastitis in Dairy Cows

**DOI:** 10.3390/pathogens9110961

**Published:** 2020-11-19

**Authors:** Noppason Pangprasit, Anyaphat Srithanasuwan, Witaya Suriyasathaporn, Surachai Pikulkaew, John K. Bernard, Wasana Chaisri

**Affiliations:** 1 Faculty of Veterinary Medicine, Chiang Mai University, Chiang Mai 50100, Thailand; panny.2.yaa@gmail.com (N.P.); numwan.sw@gmail.com (A.S.); Suriyasathaporn@hotmail.com (W.S.); surapikulkaew@gmail.com (S.P.); 2 Research Center of Producing and Development of Products and Innovations for Animal Health, Chiang Mai University, Chiang Mai 50100, Thailand; 3 Department of Animal and Dairy Science, College of Agricultural and Environmental Sciences, Tifton Campus, The University of Georgia, Tifton, GA 31793, USA; jbernard@uga.edu

**Keywords:** germicidal activities, acetic acid, lactic acid, lauric acid, caprylic acid, mastitis pathogens

## Abstract

The present study evaluated the antimicrobial activities of acetic acid against bovine mastitis pathogens compared to lactic acid and lauric and caprylic saturated fatty acids. Eleven mastitis pathogens were isolated from sub-clinical and clinical bovine mastitis cases for the study. An initial screening of their antibacterial activities by agar well diffusion method was performed. The minimum inhibitory concentration (MIC) and minimum bactericidal concentration (MBC) of each acid were obtained using a microdilution method; each acid was diluted from stock solution and then were diluted with culture broth to reach concentrations ranging from 4 to 0.004% *w/v*. The results showed acetic acid had the highest zone of inhibition against all pathogens except *Escherichia coli* compared with lauric and caprylic acids. The MIC and MBC were lowest for acetic acid against both Gram-positive (except *Staphylococcus chromogenes* from the coagulase negative staphylococci (CNS) group) and Gram-negative pathogens, intermediate for lactic and caprylic acids and greatest for lauric acid. In conclusion, acetic acid had antimicrobial activities against most mastitis pathogens compared with other acids. Further studies are needed to optimize the formulation and concentration of acetic acid for teat-dipping agent in the future.

## 1. Introduction

Mastitis, the disease causing the most economical loss in the dairy industry worldwide [1], is mostly caused by bacterial intramammary infection (IMI). Bacteria that cause mastitis are typically either contagious pathogens or environmental pathogens. During milking, environmental pathogens on the teat may cause infection prior to cluster application, while the IMI from contagious pathogens occur during and after milking when the teat channel is open [2]. Minimizing bacteria on the teat ends pre- and post-milking by dipping with a germicide has been established as the most effective procedure for mastitis prevention [2,3]. Teat dip products are chemical solutions that usually contain a blend of emollients and active germicide substance. The most commonly used germicide is iodine, which accounts for approximately 60 to 70% of the market in the USA and Europe [4]; however, iodine teat dips are classified as a medication according to FDA establishment registration and European regulations for veterinary products (EEC 2001/82). As a milk quality concern, contamination of milk with iodine is a consumer safety issue when used as a pre-dip and when cows are not wiped dry before attaching the milking unit. Therefore, teat dips based on natural products to prevent mastitis should be considered for submission to the appropriate veterinary medicine regulator for approval. Natural products used as effective germicides with lower market share include lactic acid and saturated fatty acids, e.g., lauric acid and caprylic acid [5] (Lauricidin^®^, 3M Company, St. Paul, MN, USA). Acetic acid is a colorless liquid with a sharp, distinctive odor and has the characteristic taste associated with vinegar when diluted to 20% or less with water and used to season food to enhance its taste. Acetic acid has been a widely used topical germicide agent for the treatment of burn wounds and has previously been shown to have activity against the Gram-positive organism *Staphylococcus aureus* [6] and Gram-negative organisms including *Pseudomonas aeruginosa* [7].

It is routine practice when assessing the efficacy of disinfectants to choose all bacterial pathogens causing mastitis, including minor mastitis pathogens such as coagulase negative staphylococci (CNS) species that have been associated with clinical mastitis [8]. The objective of the present study was to evaluate the antimicrobial activities of acetic acid compared with lactic, lauric and caprylic acids against bovine mastitis pathogens, including the major pathogens *Streptococcus agalactiae*, *Streptococcus uberis*, *S. aureus*, *E. coli* and *Klebsiella* spp. [9] and six minor pathogens in the CNS group. An initial screening by agar well diffusion method was performed to evaluate 20% *w/v* acetic acid on antimicrobial activities compared with 20% *w/v* for the other acids. Minimum inhibitory concentration (MIC) and minimum bactericidal concentration (MBC) among the four acids were compared.

## 2. Results

Results on inhibition zones from the agar well diffusion method of all acids against the mastitis pathogens are shown in Table 1. No differences were observed among acids in the inhibition zones for *S. agalactiae* and *S. aureus.* Acetic and lactic acids had greater (*p* < 0.05) inhibition zones for *S. uberis*, *Staphylococcus simulans* and *Staphylococcus epidermidis* compared with lauric and caprylic acid. The zone of inhibition was greatest (*p* < 0.05) for lactic acid compared with lauric and caprylic acid, but did not differ from that of acetic acid. The zones of inhibition for CNS (*Staphylococcus xylosus*, *Staphylococcus chromogenes*, *Staphylococcus hyicus* and *Staphylococcus haemolyticus*) was the greatest (*p* < 0.05) for acetic acid, intermediate for lactic acid and lowest for lauric and caprylic acids.

Due to the non-normally distributed data, logarithm transformation was applied to both MIC and MBC before performing statistical analyses. The MIC and MBC results for the tested microorganisms are shown in Table 2. All bacteria exhibited a strong tolerance to lauric acid, as indicated by the highest MIC and MBC compared with the other acids. Acetic acid exhibited the greatest (*p* < 0.05) bacteriostatic properties against both Gram-positive bacteria (*S. aureus*, *S. agalactiae*, *S. uberis*), including all CNS species except *S. chromogens*, and Gram-negative pathogens (*E. coli, Klebsiella* spp.) based on MIC values. The MBC values were lowest (*p* < 0.05) for acetic and lactic acids, intermediate for caprylic acid, and highest for lauric acid for *S. aureus*, *E. coli*, *S. epidermidis*, *S. hyicus* and *S. haemolyticus.* The MBC observed for *S. uberis* was lowest (*p* < 0.05) for acetic acid. Lactic acid has the lowest (*p* < 0.05) MBC for *S. simulans*, whereas acetic and caprylic acids were intermediate compared with lauric acid, which was highest. The MBC for *S. xylosus* was different among all acids and was lowest for acetic acid, followed by lactic acid and then caprylic acid, and highest for lauric acid.

## 3. Discussion

Bovine mastitis is considered to be one of the most economically important diseases in the dairy industry due to reduced milk yield, lowered milk quality, and increased cost for labor, drugs and veterinary services [1,10]. In a field trial, Pankey et al. [11] reported a 51% reduction in new IMI caused by streptococci and coliforms, environmental mastitis pathogens, when pre-dipping was combined with “good udder preparation”. For controlling contagious mastitis caused by *S*. *agalactiae* and *S. aureus*, the National Mastitis Council developed a mastitis control program known as the “5-Point Plan” that includes the application of an effective post-milking teat dip as one of the plan’s key points [12]. Finding a natural product for use in food production, such as acetic acid from vinegar, to use as an antimicrobial substance as a teat dip solution is an advantage for the future of the dairy industry and consumer safety.

The agar diffusion antimicrobial test has traditionally been used to evaluate the antibacterial activity of fatty acids and has the potential to be used for determining the efficacy of teat disinfectants against different types of mastitis pathogens [13,14]. The results of the zone of inhibition screen are generally in agreement with the results provided by MIC and MBC. Acetic acid had the lowest MIC and MBC for most mastitis pathogens (Table 2) and is in agreement with the zone of inhibition results (Table 1). For *S. agalactiae*, *S*. *aureus* and *S. uberis*, the MIC and MBC with acetic acid were minimal, and the MBC of lactic acid were the lowest for Gram-negative bacteria, namely *E. coli* and *Klebsiella* spp. Acetic and lactic acids had the best antimicrobial properties for CNS. All MBC values for acetic acid were equal to or less than 1%, which is supported by results from an in vitro study reporting that 3% acetic acid was an excellent bactericide against human local infection and burn-wound sepsis from *E. coli*, *P. vulgaris*, *P. aeruginosa*, *A. baumannii*, *E. faecalis*, *S. epidermidis*, methicillin-resistant *Staphylococcus aureus* (MRSA) and the β-hemolytic *Streptococcus* groups A and B or *S. agalactiae* [6]. Except for *Klebsiella* spp., the MIC and MBC of lauric acid were lower than those observed for caprylic acid. In contrast, the MIC of lauric acid against *S. aureus*, *S. agalactiae* as *Streptococcus* β-hemolytic non-A group and *S. epidermidis* were less than those of caprylic acid [15]. The antimicrobial activity of a fatty acid depends on its nature, e.g., chain length and the presence, number and position of double bonds [15,16], and the bacteria tested. Organic acids, including acetic acid or lactic acid, generally have a pKa below that of the saturated fatty acids that support higher bactericidal activity [17]. The methicillin-susceptible strain of *S. aureus* (MSSA) had an MIC of 0.312%, whereas the methicillin-resistant strain was only marginally less susceptible (MIC: 0.625%) when compared with acetic acid [18]. In addition, the use of field strains of mastitis pathogens in this study instead of reference strains might be a cause of the difference to the results previously reported.

Acetic acid has been commonly used in medicine for more than 6000 years for the disinfection of wounds and, especially, as an antiseptic agent in the treatment and prophylaxis of the plague. The results of this study indicate that acetic acid has greater germicidal activity than lactic, lauric or caprylic acids against most pathogens that cause mastitis. Further studies are needed to optimize the formulation and concentration of acetic acid, especially the combination of acetic acid and other natural products, to increase the antimicrobial effectiveness against mastitis pathogens. Field trials are needed to determine the germicidal effectiveness on mastitis prevention and effect on teat skin condition before extended use in the dairy industry.

## 4. Materials and Methods

### 4.1. Preparation of Acid Solutions

Acetic (C-2), lactic (C-3) and the saturated fatty acids—caprylic (C-8) and lauric (C-12)—used in this study were analytical grade and purchased from Sigma-Aldrich (ST. Louis, MO, USA) and Alfa Aesar (Lancashire, UK), respectively. A stock solution of each acid was prepared by dissolving 2 g of the specified acid with 8 mL of 1:1 *v/v* mixture of 50% ethanol and 99.8% dimethylsulfoxide (DMSO) to yield the final concentration of 20% *w/v*. The stock solutions were used within 24 h of preparation.

### 4.2. Selection and Preparation of Mastitis Pathogens

Mastitis pathogens used in this study included 2 reference strains of *S. aureus* ATCC25923 and *E. coli* ATCC25922 and 53 confirmed field strains of 11 mastitis pathogens collected by the Faculty of Veterinary Medicine, Chiang Mai University, as previously described [19]. Five isolates of each pathogen were selected from the stock to be the representative of pathogens including *S. agalactiae*, *S. aureus*, *S. uberis*, *E. coli*, *Klebsialla* spp. including *K. pneumoniae* and *K. variicola*, and the CNS species *S. chromogenes*, *S. epidermidis*, *S. haemolyticus*, *S. hyicus*, *S. simulans* and *S. xylosus.* These pathogens were isolated from sub-clinical and clinical bovine mastitis cases. All isolates of each selected pathogen were randomly selected from the stock, streaked onto Tryptone Soya agar (TSA; HIMEDIA, Mumbai, India) and incubated at 37 °C for 24 h. A single colony of each was transferred into Muller–Hinton broth medium (MHB; HIMEDIA, Mumbai, India). A purified bacterium was added with 800 µL of inoculum to 400 µL of 70% glycerol in a 1.5 mL cryovial tube and used for antimicrobial activity testing including the agar well diffusion method to determine MIC and MBC.

### 4.3. Agar Well Diffusion Method

The screening method used for evaluating antimicrobial activity was performed in duplicate using an agar well diffusion method as previously described by Balouiri et al. [20]. Five strains of each of the 11 pathogens isolated were suspended in 9 mL of 0.85% NSS with a concentration of 10^7^–10^8^ CFU/mL (McFarland density = 0.5) and uniformly spread using a sterile cotton swab on a TSA. Thirty-five microliters of each prepared acid treatment was added to each agar well prepared by cutting a 6 mm diameter hole in the agar gel 15 to 20 mm apart from one another. Agar wells were incubated at 37 °C for 24 h before evaluation. The antimicrobial activity was evaluated by the appearance of the inhibitory zone around the agar plug. The inhibitory zone was measured in mm using Vernier Calipers [21]. Experiments were performed in duplicate.

### 4.4. Determination of MIC and MBC

The MIC and MBC of the prepared mastitis pathogens were obtained using a microdilution method, following Clinical Laboratory and Standard Institute guidelines [22]. Each acid was diluted from stock solution in a solvent containing 50% ethanol 1:1 DMSO (80 mg/mL of the final concentration) and then diluted with culture broth to a concentration of 4% *w/v*. Further, two serial dilutions were performed by addition of culture broth to reach concentrations ranging from 4 to 0.004% *w/v* using 96-well plates. Each well was inoculated with 10 μL of bacterial suspension to yield a final inoculum concentration of 10^5^ CFU/mL and then incubated at 37 °C for 24 h. The MIC and MBC evaluations were performed in duplicate for each combination of bacteria and acid. The lowest concentration which inhibited visible growth of bacteria in broth was defined as the MIC. For MBC, 10 μL of transparent medium were smeared on TSA and incubated at 37 °C for 24 h. The lowest acid concentration that inhibited the growth of bacteria on agar medium was defined as the MBC.

### 4.5. Statistical Analysis

Results of antimicrobial efficiencies, including growth inhibition zone diameters and the lowest concentrations of MIC and MBC, of the acids among specific pathogens were described as means ± standard error of the mean (SEM). The antimicrobial efficiencies data were tested for their normal distribution for each pathogen. Logarithm transformation was applied for non-normally distributed data. Differences in growth inhibition zone, MIC and MBC among the four acids for each pathogen were calculated by applying the one-way analysis of variance (ANOVA), and Tukey’s multiple-range tests, SAS^®^ University Edition, were used for pairwise comparison. Significance was defined as *p* < 0.05.

## 5. Conclusions

Acetic acid exhibited antimicrobial activities against most mastitis pathogens compared with other acids. Further studies are needed to optimize the formulation and concentration of acetic acids, especially regarding its combination with other natural products, as effective natural teat-dipping agents.

## Figures and Tables

**Table 1:** Mean and standard error of the mean (SEM) of inhibition zones of acetic, lactic, lauric and caprylic acids at 20% *w/v* against mastitis pathogens using agar well diffusion methods.

	Acetic	Lactic	Lauric	Caprylic
*S. agalactiae*	36.0 + 5.42	34.0 + 2.17	27.8 + 3.59	30.2 + 5.29
*S. aureus*	24.6 + 3.40	21.2 + 0.49	17.6 + 1.86	16.4 + 1.75
*S. uberis*	33.4 + 1.08 ^a^	28.2 + 1.98 ^a^	21.8 + 1.98 ^b^	17.6 + 0.68 ^b^
*E. coli* ^2^	19.55 + 2.90 ^a^	24.0 + 1.91 ^a^	12.2 + 0.25 ^b^	12.5 + 0.29 ^b^
*Klebsiella spp.*	31.7 + 3.84 ^a^	25.0 + 1.08 ^ab^	11.0 + 0.00 ^b^	13.0 + 0.00 ^ab^
*S. simulans* ^1^	33.3 + 0.88 ^a^	30.0 + 1.15 ^a^	19.7 + 1.45 ^b^	18.0 + 0.00 ^b^
*S. epidermidis* ^1^	28.3 + 2.03 ^a^	26.0 + 1.15 ^a^	16.7 + 0.88 ^b^	16.3 + 0.33 ^b^
*S. xylosus* ^1^	43.6 + 0.60 ^a^	34.8 + 0.86 ^b^	18.0 + 1.82 ^c^	20.0 + 0.55 ^c^
*S. chromogenes* ^1,2^	40.0 + 2.77 ^a^	30.6 + 1.47 ^b^	16.0 + 1.09 ^c^	15.4 + 0.40 ^c^
*S. hyicus* ^1,2^	42.8 + 1.49 ^a^	31.2 + 1.83 ^ab^	22.6 + 3.56 ^b^	23.2 + 4.86 ^b^
*S. haemolyticus* ^1^	37.2 + 0.86 ^a^	24.4 + 1.17 ^b^	17.8 + 1.74 ^c^	19.4 + 2.06 ^bc^

^a–c^ Means in the same row with different superscripts differ (*p* < 0.05). ^1^ Bacteria in the coagulase negative staphylococci (CNS) group. ^2^ Logarithm transformation due to data being non-normal before statistical analysis.

**Table 2:** Mean and standard error of the mean (SEM) of minimum inhibitory concentration (% *w/v*) and minimum bactericidal concentration (% *w/v*) of acetic, lactic, lauric and caprylic acids against mastitis pathogens.

	Minimum Inhibitory Concentration (MIC)				Minimum Bactericidal Concentration (MBC)			
	Acetic	Lactic	Lauric	Caprylic	Acetic	Lactic	Lauric	Caprylic
*S. agalactiae*	0.125 + 0.00 ^c^	0.25 + 0.00 ^b^	0.88 + 0.12 ^a^	0.22 + 0.03 ^b^	0.25 + 0.00 ^c^	0.25 + 0.00 ^c^	4.00 + 0.00 ^a^	0.56 + 0.16 ^b^
*S. aureus*	0.125 + 0.00 ^c^	0.42 + 0.05 ^b^	4.00 + 0.00 ^a^	0.50 + 0.00 ^b^	0.45 + 0.04 ^c^	0.50 + 0.00 ^c^	4.00 + 0.00 ^a^	1.00 + 0.00 ^b^
*S. uberis*	0.125 + 0.00 ^d^	0.50 + 0.00 ^b^	1.00 + 0.00 ^a^	0.25 + 0.00 ^c^	0.25 + 0.00 ^c^	0.50 + 0.00 ^b^	2.00 + 0.00 ^a^	0.50 + 0.00 ^b^
*E. coli*	0.125 + 0.00 ^d^	0.30 + 0.05 ^c^	2.00 + 0.00 ^a^	1.00 + 0.00 ^b^	0.65 + 0.15 ^b^	0.60 + 0.10 ^b^	4.00 + 0.00 ^a^	2.40 + 0.40 ^a^
*Klebsiella* spp.	0.125 + 0.00 ^c^	0.29 + 0.04 ^b^	1.00 + 0.00 ^a^	1.00 + 0.00 ^a^	0.50 + 0.00 ^b^	0.50 + 0.00 ^b^	4.00 + 0.00 ^a^	4.00 + 0.00 ^a^
*S. simulans* ^1^	0.225 + 0.02 ^c^	0.45 + 0.05 ^b^	2.00 + 0.00 ^a^	0.50 + 0.00 ^b^	1.00 + 0.00 ^b^	0.50 + 0.00 ^c^	4.00 + 0.00 ^a^	1.40 + 0.24 ^b^
*S. epidermidis* ^1^	0.125 + 0.00 ^d^	0.25 + 0.00 ^c^	2.00 + 0.00 ^a^	0.50 + 0.00 ^b^	0.50 + 0.00 ^c^	0.50 + 0.00 ^c^	4.00 + 0.00 ^a^	1.80 + 0.20 ^b^
*S. xylosus* ^1^	0.15 + 0.02 ^d^	0.25 + 0.00 ^c^	2.00 + 0.00 ^a^	0.50 + 0.00 ^b^	0.25 + 0.00 ^d^	0.50 + 0.00 ^c^	4.00 + 0.00 ^a^	2.00 + 0.00 ^b^
*S. chromogenes* ^1^	0.25 + 0.12 ^b^	0.25 + 0.00 ^b^	1.50 + 0.29 ^a^	0.28 + 0.12 ^b^	0.44 + 0.06 ^b^	0.44 + 0.06 ^b^	3.50 + 0.50 ^a^	0.41 + 0.09 ^b^
*S. hyicus* ^1^	0.125 + 0.00 ^c^	0.40 + 0.06 ^b^	2.00 + 0.00 ^a^	0.60 + 0.10 ^b^	0.50 + 0.00 ^c^	0.45 + 0.05 ^c^	4.00 + 0.00 ^a^	1.20 + 0.20 ^b^
*S. haemolyticus* ^1^	0.125 + 0.00 ^c^	0.25 + 0.00 ^b^	2.00 + 0.00 ^a^	0.30 + 0.05 ^b^	0.25 + 0.00 ^c^	0.25 + 0.00 ^c^	4.00 + 0.00 ^a^	0.70 + 0.12 ^b^

^a–d^ Means in the same row with different superscripts differ (*p* < 0.05). ^1^ Bacteria in the coagulase negative staphylococci (CNS) group.
